# GA-TAN: a graph aggregation and topology-aware network for unified 3D ocular–cerebral vascular segmentation

**DOI:** 10.3389/fmed.2026.1812113

**Published:** 2026-04-02

**Authors:** Jianming Zheng, Zihan Wang, Jinxian Zhang, Jianing Ying, Tao Chen, Quanyong Yi, Jiong Zhang

**Affiliations:** 1 Laboratory of Advanced Theranostic Materials and Technology, Ningbo Institute of Materials Technology and Engineering, Chinese Academy of Sciences, Ningbo, China; 2 University of Chinese Academy of Sciences, Beijing, China; 3 The Affiliated Ningbo Eye Hospital, Ningbo University, Ningbo, China; 4 Ningbo Eye Hospital, Wenzhou Medical University, Ningbo, China; 5 Eindhoven University of Technology, Eindhoven, Netherlands; 6 Ningbo Cixi Institute of Biomedical Engineering, Cixi, China

**Keywords:** cerebrovasculature, CNN, ocular, transformer, VIG

## Abstract

**Background:**

The eye and brain share developmental and regulatory vascular similarities, and 3D vascular segmentation from Optical Coherence Tomography Angiography (OCTA) and Magnetic Resonance Angiography (MRA) is essential for neuro-ophthalmic assessment. However, vascular trees exhibit extreme multi-scale morphology and are vulnerable to topology disruptions caused by OCTA artifacts (e.g., noise and discontinuities) and MRA anatomical variants (e.g., Circle of Willis incompleteness), which often lead to fragmented vessels and unstable segment-wise predictions.

**Purpose:**

To develop a unified 3D vascular segmentation framework that transfers and shares structural priors across OCTA and MRA, enabling accurate parsing under extreme scale imbalance and topology disruptions, and producing anatomically plausible predictions that remain robust to imaging artifacts in OCTA and Circle-of-Willis variants in MRA.

**Methods:**

We propose GA-TAN, a Graph Aggregation and Topology-Aware Network for unified ocular–cerebral vascular segmentation. GA-TAN employs a variable-window hybrid CNN–Transformer backbone to adapt receptive fields across vessel scales, an Efficient Multi-Scale Attention (EMA) module to enhance volumetric spatial–channel interactions, and a Multi-Scale Graph Aggregation (MSGA) module to perform global topological reasoning and hierarchical feature fusion. A topology-aware training objective further supervises segment existence and connectivity to penalize structural discontinuities beyond pixel-wise losses. Experiments were conducted on Retina3D for binary 3D OCTA vessel segmentation and on TopCow for 13-class CoW artery segmentation from MRA.

**Results:**

GA-TAN achieved the best overall performance on Retina3D, with a DSC of 0.684 and a clDice of 0.667, indicating improved overlap accuracy and centerline connectivity preservation. On TopCow, GA-TAN obtained a mean Dice of 0.814 across 13 arterial categories and produced more anatomically consistent segment-wise predictions, showing clear advantages on thin communicating and variant-related segments. Ablation studies further validated the complementary contributions of EMA, MSGA, and topology-aware supervision.

**Conclusion:**

GA-TAN provides a unified and topology-aware solution for 3D vascular segmentation across OCTA and MRA. By integrating adaptive multi-scale modeling, graph-based global aggregation, and explicit topology supervision, the proposed framework improves both segmentation accuracy and vascular ontinuity, enhancing robustness to imaging artifacts and anatomical variants for ocular–cerebral vascular analysis.

## 1 Introduction

The brain and the eye are two tightly coupled organs with shared developmental origins and systemic vascular regulation. As a consequence, retinal microvascular alterations are increasingly recognized as accessible surrogate markers for cerebrovascular health, enabling potential early screening and risk stratification of vascular-related diseases. In clinical practice, optical coherence tomography angiography (OCTA) provides non-invasive three-dimensional (3D) visualization of retinal microvasculature, while magnetic resonance angiography (MRA) depicts intracranial arterial anatomy such as the Circle of Willis (CoW). Recent OCTA studies have also explored wide-field reconstruction and image stitching to alleviate the limited field of view of commercial devices, further highlighting the growing importance of OCTA for large-scale retinal vascular analysis (1). A unified analysis of ocular and cerebral vasculature is therefore of growing interest, with OCT/OCTA-based retinal vascular imaging continuing to attract substantial attention in ophthalmic research (2). However, accurate automated vessel segmentation across these two 3D modalities remains challenging.

**Figure 1:**
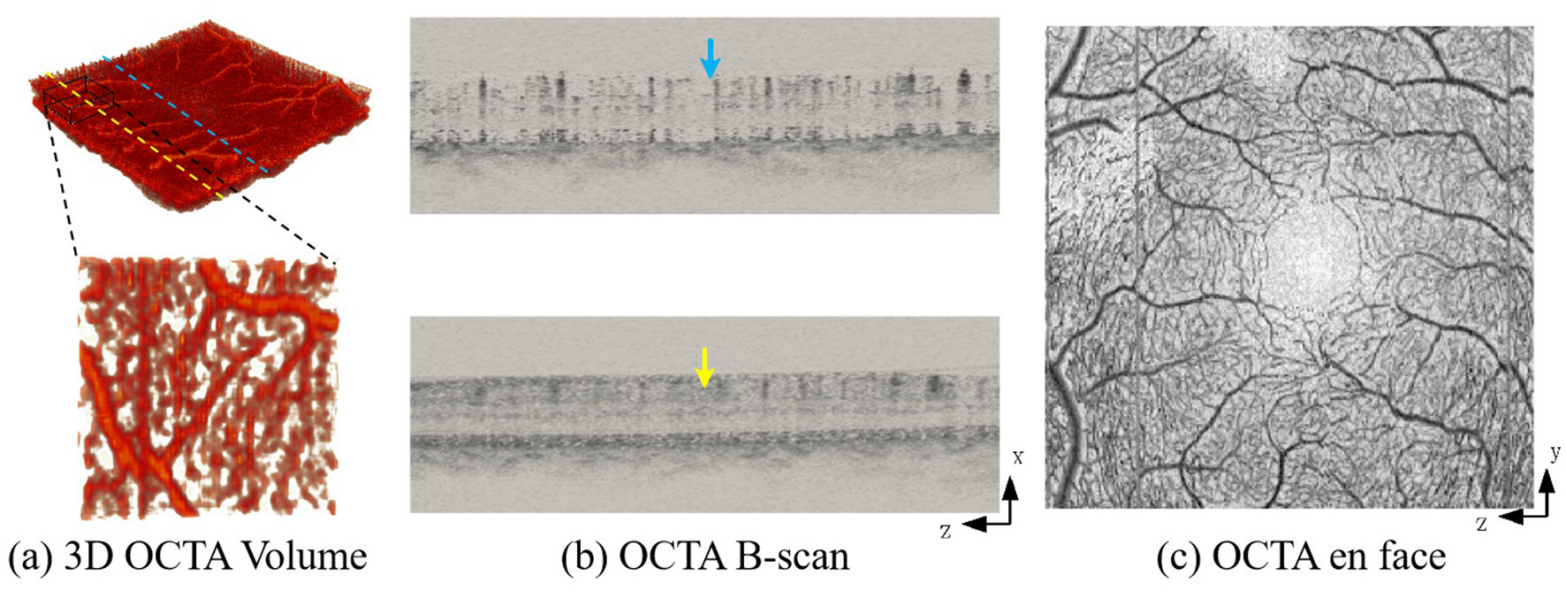
Visualization of the structural components of OCTA data. **(a)** A 3D OCTA volumetric rendering showing the spatial arrangement of retinal vessels. The blue and yellow dashed lines indicate different depth planes (e.g., superficial and deep vascular plexuses), with the zoomed-in block highlighting fine capillary details. **(b)** Corresponding cross-sectional OCTA B-scans at the depths marked by the blue and yellow arrows, demonstrating flow signals within specific retinal layers. **(c)** A 2D en face projection image. While widely used, this projection collapses the volumetric depth information shown in **(a, b)** into a single plane, potentially obscuring complex 3D topological relationships.

Despite both being volumetric, OCTA and MRA exhibit distinct imaging characteristics and failure modes. OCTA captures dense capillary plexuses with distinct layer-wise organization and enables non-invasive 3D visualization of retinal microvasculature through repeated cross-sectional acquisitions (3). As illustrated in Figure 1, the 3D vascular volume (Figure 1a) is constructed from cross-sectional B-scans (Figure 1b) that reveal flow signals at varying depths (indicated by blue and yellow arrows). However, reliable 3D vessel extraction is often hindered by limited capillary visibility, complex vascular topology, motion artifacts, and speckle noise, which jointly cause discontinuities, spurious connections, and ambiguous boundaries for thin vessels. Moreover, many OCTA pipelines still rely on 2D en-face projections (Figure 1c) for convenience, inevitably discarding the critical depth-wise information visible in the B-scans and weakening the exploitation of full volumetric context. Together with the scarcity of manually annotated 3D OCTA data, these factors substantially limit the robustness and clinical reliability of automated microvascular quantification.

**Figure 2:**
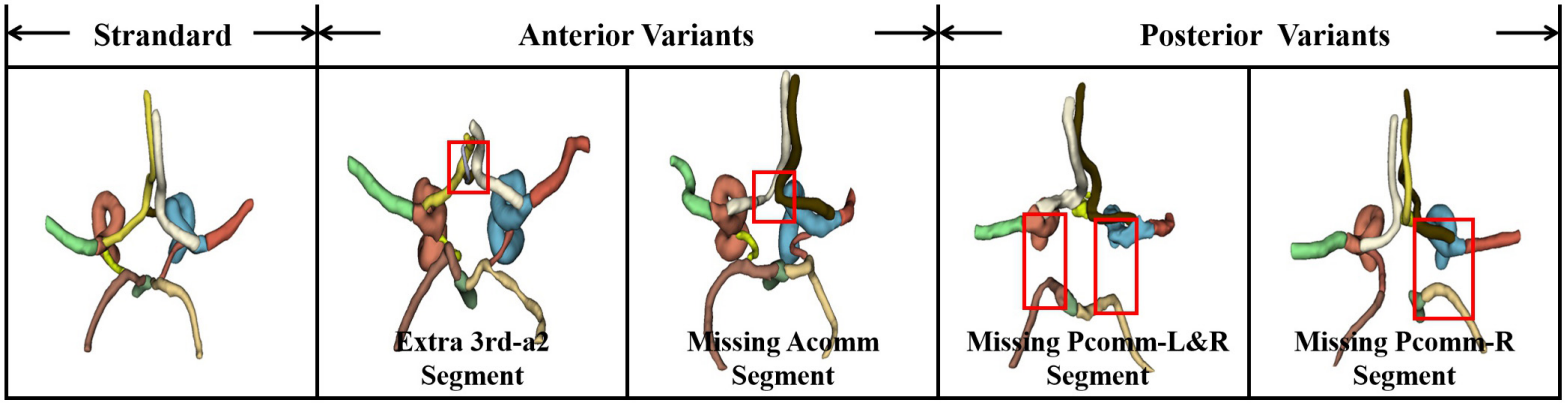
Illustration of topological variations in the Circle of Willis (CoW) from MRA data. The leftmost panel displays the standard CoW structure. The subsequent panels demonstrate common anterior and posterior variants, including extra segments (e.g., 3rd-A2), missing Anterior Communicating Arteries (Acomm), and missing Posterior Communicating Arteries (Pcomm). Red boxes highlight the specific regions where the topology deviates from the standard pattern, posing challenges for automated connectivity analysis.

In contrast, cerebral MRA typically suffers from limited spatial resolution and intensity inhomogeneity, and intracranial arteries form complex multi-branch structures with pronounced inter-subject variability. Notably, only a small proportion of individuals present a complete CoW, whereas most cases exhibit topological variants such as aplasia, hypoplasia, or duplication of arterial segments (4). As shown in Figure 2, these structural anomalies range from anterior variants (e.g., extra segments or missing Anterior Communicating Artery) to posterior variants (e.g., missing Posterior Communicating Arteries). These variants, highlighted by the red boxes in Figure 2, significantly alter the vascular topology compared to the standard model. Such deviations break canonical connectivity patterns and often induce systematic class assignment errors, especially for short or low-contrast segments. Although deep learning has advanced intracranial multi-class vessel segmentation (5), existing models still show a clear performance gap between large trunks and thin/short segments under severe class and scale imbalance, and they remain fragile when topology deviates from canonical patterns (6).

These observations highlight common core difficulties shared by 3D OCTA and 3D MRA vascular analysis: (i) multi-scale vessel morphology with extreme imbalance between large vessels and thin/short segments, and (ii) topology disruptions caused by either imaging artifacts (e.g., breaks and spurious connections in OCTA) or anatomical variants (e.g., missing/duplicated segments in the CoW). Therefore, an effective 3D eye–brain vascular framework should jointly model fine-grained local details, long-range contextual dependencies, and explicit topology constraints.

To address these challenges, we propose GA-TAN, a Graph Aggregation and Topology-Aware Network that integrates multi-level feature aggregation for robust vascular segmentation. GA-TAN adopts a hybrid CNN–Transformer backbone to simultaneously capture local vessel details and global morphology. We further design: (1) a variable window mechanism to enable multi-scale contextual modeling and improve sensitivity to thin vessels and short segments; (2) an efficient multi-scale attention (EMA) module to enhance spatial–channel interactions and strengthen topology-related representations; (3) a multi-scale graph aggregation (MSGA) module to promote cross-level feature fusion and mitigate the imbalance of vessel segment proportions; and (4) a topology-aware loss to explicitly supervise segment existence and connectivity, reducing errors induced by topology disruptions and anatomical variants. We validate GA-TAN on the public TopCow dataset (7), achieving superior accuracy and robustness compared with state-of-the-art methods.

In summary, our main contributions are:

1. We propose a variable window mechanism with multi-channel decoupling to capture multi-scale context and improve sensitivity to thin vessels and short segments under severe imbalance.
2. We design an efficient multi-scale attention module to enhance spatial–channel interaction and strengthen topology-related feature representation.
3. We develop a multi-scale graph aggregation module that performs hierarchical graph-based feature fusion, improving multi-scale vascular perception and mitigating imbalance across vessel segments.
4. We introduce a topology-aware loss that supervises vessel existence and connectivity, reducing errors induced by topological disruptions and anatomical variants.

## 2 Related work

### 2.1 Vascular segmentation in OCTA and MRA

Accurate segmentation of vascular structures is a prerequisite for quantitative analysis in both ophthalmology and neurology. Previously, segmentation methods for OCTA were primarily based on 2D en-face projections (8–10), aimed at reducing the computational burden and avoiding the complexity of volumetric data. For instance, projection-based networks (9) and attention-enhanced variants (10) have been developed to learn discriminative vessel features in a projected 2D space. A deep learning-based automated OCTA analysis framework has also been developed for quantifying retinal microvascular alterations associated with Alzheimer's disease and mild cognitive impairment, further demonstrating the clinical relevance of reliable vessel segmentation in en-face OCTA analysis (8). More recently, topology-sensitive OCTA methods have started to exploit structural priors beyond generic semantic encoding. A direction-guided network has been proposed to enhance thin-vessel detection and reconnect fragmented capillaries through directional convolution and direction-aware feature aggregation, highlighting the importance of directional and connectivity cues in OCTA vessel segmentation (11). However, these approaches inevitably discard critical depth-wise information and fail to capture the complex 3D topological relationships of the capillary plexuses. To address this, recent studies have shifted toward direct 3D segmentation (12–14), employing volumetric architectures to preserve spatial continuity. A topology-aware 3D OCTA microvascular segmentation framework has further been introduced to explicitly suppress motion artifacts and reinforce vessel continuity through multi-view feature learning, highlighting the importance of topology preservation in retinal OCTA analysis (14). Despite these advances, 3D OCTA segmentation remains challenging; the inherent speckle noise and projection artifacts often lead to vessel discontinuities and ambiguous boundaries, making it difficult for standard CNNs to maintain topological correctness.

Similarly, in the realm of cerebral MRA, deep learning approaches have largely superseded traditional filtering techniques. While standard architectures like V-Net (15) and self-configuring frameworks like nnU-Net (16) establish strong baselines, they often struggle with the extreme scale imbalance between large arterial trunks and thin distal vessels. To capture long-range dependencies, Transformer-based models such as UNETR and Swin UNETR have been introduced (17, 18). Recent studies have further emphasized the necessity of preserving vascular continuity in cerebrovascular segmentation. A connectivity-reinforced framework for TOF-MRA has been proposed by combining a connectivity attention module with an adaptive connectivity loss to alleviate disconnected and incomplete vessel predictions (19). In parallel, a fully automated 3D cerebrovascular segmentation workflow has been developed across bright- and black-blood MRA sequences, showing that transformer-based models such as SwinUNETR provide strong performance for vessels of varying calibers (20). It is worth noting that, although these methods improve global morphological modeling, they are still fragile when handling anatomical variants (e.g., anomalies in the Circle of Willis) (7). The topological disruptions caused by these variants or imaging artifacts frequently result in systematic class assignment errors in multi-class segmentation, especially under anatomical variants. Therefore, developing a unified framework capable of handling multi-scale vascular morphology while enforcing topological consistency remains a significant challenge.

### 2.2 Graph-based and topology-aware learning

In recent years, Graph Convolutional Networks (GCNs) have gained significant attention in medical image analysis for their ability to model non-Euclidean relationships. By processing graph-structured data, GCNs can effectively capture global contextual information and reason about connectivity, achieving state-of-the-art performance in vascular analysis tasks (21, 22).

Specifically, graph convolutions in this domain can be generally categorized into two types: *coordinate space graph convolution* and *feature space graph convolution*. The former models spatial relationships explicitly, where nodes correspond to spatial entities like skeleton points or superpixels (23). This approach is advantageous for preserving geometric connectivity but often lacks flexibility in high-level semantic reasoning. The latter, feature space graph convolution, projects feature maps into a latent interaction space to capture long-range interdependencies across channels or regions (24). A multilateral interaction-enhanced graph network has recently been proposed to construct task-specific graphs for reasoning about region, boundary, and shape constraints, demonstrating the effectiveness of feature-space interaction in resolving ambiguous boundaries (25). Related developments have further extended graph learning beyond single-modality representation, showing that graph structures can also serve as effective carriers for cross-modal interaction and fine-grained relational reasoning. A cross-modal graph learning framework for PVS segmentation has been introduced, in which textual priors guide image feature extraction and graph reasoning enhances multimodal interaction, highlighting the potential of graph-based models for capturing subtle structures under complex morphology and low contrast (26). In retinal vessel analysis, graph convolutional attention has further been combined with a multi-scale vision transformer, demonstrating that graph-enhanced feature interaction can improve the segmentation of fine vascular branches and complement hierarchical contextual modeling (27).

However, most existing methods treat segmentation and topology as separate concerns or rely solely on pixel-wise supervision. This limitation is especially relevant in retinal vessel analysis, where existing deep learning-based methods still struggle with fine vessel segmentation and often rely heavily on time-consuming pixel-level annotations (28). To address the connectivity issues prevalent in both OCTA (e.g., noise-induced breaks) and MRA (e.g., variant-induced gaps), topology-aware learning strategies have been explored. Loss functions based on centerline continuity or persistent homology have been designed to penalize topological errors explicitly (29, 30). Beyond explicit topology losses, recent work has also shown that topology-aware optimization can be embedded into the training process itself. A meta-tubular robust learning framework has been proposed to decompose retinal vessels into tubular compartments and design tubular-wise reweighting and loss functions, thereby improving robustness to noisy annotations while preserving fine vessel topology (31). Building on these insights, our proposed GA-TAN integrates feature-space graph aggregation with explicit topology-aware constraints. Unlike previous works that focus on either local extraction or global reasoning in isolation, our method jointly models multi-scale aggregation and topological consistency, thereby enhancing robustness against both imaging artifacts in OCTA and anatomical variations in MRA.

## 3 Proposed method

**Figure 3:**
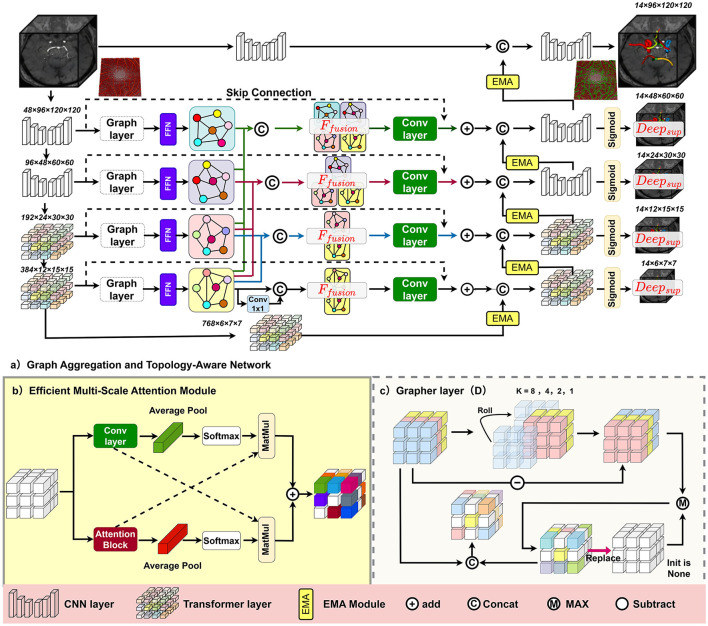
The architecture of GA-TAN. The framework is designed to process generic 3D vascular volumes (OCTA or MRA). **(a)** The backbone utilizes a variable window strategy. **(b)** The EMA module enhances 3D spatial-channel interactions. **(c)** The MSGA module constructs graph representations to reason about global connectivity and mitigate vessel scale imbalance.

We propose GA-TAN, a unified framework designed to address the shared challenges in 3D vascular analysis for both the eye and the brain: specifically, the extreme multi-scale nature of vascular trees (ranging from large intracranial arteries to microscopic retinal capillaries) and the sensitivity of topological connectivity to imaging artifacts and anatomical variants. As illustrated in Figure 3, GA-TAN consists of three main components: a variable window structure for multi-scale receptive field adaptation, an Efficient Multi-Scale Attention (EMA) module for volumetric feature refinement, and a Multi-Scale Graph Aggregation (MSGA) module for global topological reasoning. Additionally, we introduce a topology-aware training objective to explicitly enforce structural continuity and anatomical correctness.

### 3.1 Variable window structure

To model vascular structures with highly variable diameters and elongated 3D morphology, we introduce a variable window mechanism in the hybrid backbone. Unlike standard fixed-window self-attention [e.g., Swin Transformer (32)], which applies the same cubic window to all channels, the proposed design partitions the feature channels into multiple groups and assigns each group a different 3D window shape.

Given an input feature map, we first apply a shared linear projection to obtain the QKV representation. The projected channels are then evenly divided into four channel groups. Each group performs self-attention independently under its own window configuration. After the four branch-wise attention operations, their outputs are concatenated along the channel dimension and fused by a shared linear projection, followed by residual connection and feed-forward transformation. In this way, different channel groups are allowed to model different spatial dependency patterns, rather than sharing the same receptive-field geometry.

The first channel group uses a local cubic window to capture compact volumetric context. This branch mainly models short-range 3D interactions around vessel structures. The remaining three channel groups use anisotropic stripe-like windows with different orientations. Specifically, one branch adopts a depth–width window that spans the full height axis, one adopts a depth–height window that spans the full width axis, and one adopts a height–width window that spans the full depth axis. These three branches therefore introduce directional long-range interactions along the three principal spatial axes. As a result, the local cubic branch and the three stripe branches jointly encode both compact 3D neighborhoods and axis-oriented extended context.

The stripe dimensions are defined in a stage-dependent manner. In shallow encoder stages, where the feature map has relatively large spatial resolution, stripe windows preserve long-range directional dependencies while restricting the token number within each branch. As the network goes deeper and the feature map becomes smaller, the effective stripe coverage gradually increases and approaches global interaction over the reduced feature space. Therefore, the receptive field changes not only across stages, but also across channel groups within the same stage.

To enable information exchange across neighboring windows, adjacent transformer blocks alternate between regular window partitioning and shifted window partitioning. In the shifted setting, each window is cyclically displaced by half the window size along each axis. An attention mask is then introduced to restrict invalid interactions between non-neighboring regions. This shifted-window operation allows adjacent windows to communicate across block boundaries while preserving the locality of window-based attention.

Under this formulation, the variable window structure consists of one cubic attention branch and three axis-oriented stripe attention branches operating on four decoupled channel groups. The cubic branch is used to preserve local volumetric structure, whereas the stripe branches extend the receptive field along different spatial directions. The outputs of these four branches are merged after attention, providing a mixed representation of local and directional context at each stage of the encoder.

### 3.2 Efficient multi-scale attention module

Volumetric medical images, whether OCTA or MRA, often suffer from modality-specific noise (e.g., speckle noise in OCTA, intensity inhomogeneity in MRA). To robustly extract vascular features from these noisy 3D volumes, we propose the Efficient Multi-Scale Attention (EMA) module. As shown in Figure 3b, this module integrates multi-scale feature extraction with attention mechanisms to refine spatial representations.

We incorporate the Coordinate Attention Block (33), leveraging 1 × 1 × 1 convolutions along three orthogonal axes (X, Y, Z). This compresses channel dimensions to establish joint spatial-channel attention, enabling the capture of long-range dependencies across the 3D volume. To further accommodate the geometric complexity of vascular trees, we integrate a parallel 3 × 3 × 3 convolutional branch. This expands the local receptive field, ensuring that the continuity of tortuous vessels is preserved. Finally, global average pooling is applied to both the coordinate attention feature *F*_*ca*_ and the convolutional branch feature *F*_*c*_ to extract channel-wise statistics. Cross-branch feature interaction is achieved by element-wise summation, enhancing synergistic feature representation while preserving the precise spatial localization required for thin vessel segmentation.

### 3.3 Multi-scale graph aggregation module

Local convolutional features often struggle to maintain topological correctness in the presence of signal dropouts (common in OCTA) or anatomical gaps (common in MRA variants). To address this, we propose the Multi-Scale Graph Aggregation (MSGA) module, specifically designed to perform fine-grained vascular parsing through global reasoning.

As shown in Figure 3c, we design a level-specific Grapher module that dynamically maps volumetric features into graph-structured representations via Max-related Graph Convolution. By constructing a graph *G*_*i*_ at the *i*^*th*^ layer based on connections across Depth, Height, and Width, the model transcends local pixel neighborhoods to model long-range vascular connectivity. To fully exploit hierarchical information, we perform cross-layer concatenation between the graph structure *G*_*i*_ and features from subsequent layers *j* (*j*>*i*). This effectively fuses semantic strength from deeper layers with the detailed spatial resolution of shallower layers. Specifically, we apply a 1 × 1 × 1 convolution to the bottom-level graph features to align dimensions before concatenation, followed by a 3 × 3 × 3 refinement convolution. This residual aggregation mechanism ensures that faint vascular signals—often lost during downsampling—are preserved and reinforced by global context.

### 3.4 Topology-aware loss

Standard voxel-wise loss functions (e.g., Cross-Entropy) are often insufficient for vascular analysis, as they mainly optimize local classification accuracy but do not explicitly constrain structural continuity or anatomical plausibility. To address this limitation, we formulate a topology-aware loss that combines voxel-level supervision with higher-level structural constraints. Although the overall design principle is shared across modalities, the specific forms of the structural terms are adapted to the annotation granularity of each task: segment-wise anatomical priors for MRA and continuity-oriented structural priors for OCTA.

#### 3.4.1 Voxel-wise segmentation loss

We first define a voxel-wise segmentation loss $L_{pixel}$ to provide basic local supervision. Let *z*_*i, c*_ denote the logit at voxel *i* for class *c*, and let *y*_*i*_ be the corresponding ground-truth label. We define $L_{pixel}$ as the voxel-wise cross-entropy loss:


$$\begin{array}{l} L_{pixel}=-\frac{1}{N}\underset{i\in \left\{y_{i}\neq 0\right\}}{\sum }\log\left(\frac{\exp\left(z_{i,y_{i}}\right)}{\sum_{c=0}^{C-1}\exp\left(z_{i,c}\right)}\right), \end{array}$$ (1)

where *C* is the number of classes and *N* is the number of valid (non-background) voxels. For Retina3D, this reduces to the binary vessel-versus-background case, while for TopCow it corresponds to the multi-class arterial segmentation setting.

#### 3.4.2 Existence supervision

We introduce an existence loss $L_{exist}$ to provide higher-level supervision on the presence or absence of vascular structures. Since the two tasks considered in this work have different label spaces, this term is instantiated differently for MRA and OCTA.

For **MRA**, where each class corresponds to a specific named arterial segment, we adopt a *segment-wise existence* strategy. For each sample, we construct a binary existence vector indicating whether each arterial category is present in the ground truth. The model predicts a corresponding existence logit for each class by applying global average pooling over the spatial dimensions. Let *g*_*n, c*_ denote the global pooled response for class *c* in sample *n*, and let *e*_*n, c*_∈{0, 1} denote the corresponding ground-truth existence label. The existence loss is defined as:


$$\begin{array}{l} L_{exist}^{\text{MRA}}=-\frac{1}{BC}\overset{B}{\underset{n=1}{\sum }}\overset{C-1}{\underset{c=0}{\sum }}[e_{n,c}\log\sigma (g_{n,c})\\ \;+\left(1-e_{n,c}\right)\log\left(1-\sigma \left(g_{n,c}\right))\right], \end{array}$$ (2)

where *B* is the batch size, *C* is the number of classes, and *σ*(·) denotes the sigmoid function. This term explicitly encourages the network to determine whether a specific arterial segment exists, which is especially important in the presence of anatomical variants such as aplasia or hypoplasia.

For **OCTA**, the task is binary vessel segmentation with only one foreground class, so vessel-segment-specific existence modeling is not applicable. Instead, we adopt a *region-wise existence* strategy. Specifically, the OCTA volume is partitioned into *R* local subvolumes, and for each subvolume we define a binary existence label *r*_*n, k*_∈{0, 1} indicating whether vessel foreground is present in region *k* of sample *n*. Let *q*_*n, k*_ denote the predicted existence logit for the corresponding region. The OCTA existence loss is then defined as:


$$\begin{array}{l} L_{exist}^{\text{OCTA}}=-\frac{1}{BR}\overset{B}{\underset{n=1}{\sum }}\overset{R}{\underset{k=1}{\sum }}[r_{n,k}\log\sigma (q_{n,k})\\ \;+\left(1-r_{n,k}\right)\log\left(1-\sigma \left(q_{n,k}\right))\right], \end{array}$$ (3)

where *R* is the number of local sub-volumes. In this way, $L_{exist}$ in OCTA acts as a coarse structural prior that encourages the network to preserve local vessel occupancy rather than relying solely on voxel-wise classification.

For simplicity, the final existence term is denoted uniformly as $L_{exist}$, with its concrete form determined by the task.

#### 3.4.3 Connectivity supervision

We further introduce a connectivity loss $L_{conn}$ to explicitly regularize vascular connectivity. Similar to the existence term, its concrete form depends on the task.

For **MRA**, we adopt an *adjacency-guided connectivity* strategy based on anatomical priors. Since the Circle of Willis consists of named arterial segments with well-defined connectivity patterns, we construct a predefined adjacency graph that specifies which vessel pairs are anatomically allowed to connect. Let $E_{n}$ denote the set of valid vessel connection pairs in sample *n*, and let $P_{a}^{\left(n\right)}$ and $P_{b}^{\left(n\right)}$ denote the predicted softmax probability maps of vessel classes *a* and *b*, respectively. Using soft probability maps rather than hard argmax labels enables differentiable structural supervision. The connectivity loss is formulated as:


$$\begin{array}{l} L_{conn}^{\text{MRA}}=\frac{1}{B}\overset{B}{\underset{n=1}{\sum }}\underset{(a,b)\in E_{n}}{\sum }||\max(0,P_{a}^{(n)}⊙P_{b}^{(n)}\\ \;-\text{Dilate}(P_{a}^{(n)}⊙P_{b}^{(n)},K)){||}_{1}, \end{array}$$ (4)

where ⊙ denotes element-wise multiplication, *K* is a 3D structuring kernel, and Dilate(·, *K*) denotes morphological dilation. This term penalizes structurally implausible inter-segment interactions and encourages the predicted arterial arrangement to remain consistent with known vascular anatomy.

For **OCTA**, no class-level adjacency graph is available because only one foreground vessel class is annotated. Therefore, instead of explicit segment-to-segment connectivity modeling, we use a *continuity-preserving structural regularization* term. Let P and G denote the predicted vessel probability map and the ground-truth vessel mask, respectively. Let *S*(·) denote a differentiable soft-skeletonization operator. We adopt a soft clDice-style connectivity loss:


$$\begin{array}{l} \text{clDice}=\frac{2T_{\text{prec}}T_{\text{sens}}}{T_{\text{prec}}+T_{\text{sens}}}, \end{array}$$ (5)

where


$$\begin{array}{l} T_{\text{prec}}=\frac{\left|S\left(P\right)\cap G\right|}{\left|S\left(P\right)\right|},\;T_{\text{sens}}=\frac{\left|S\left(G\right)\cap P\right|}{\left|S\left(G\right)\right|}. \end{array}$$ (6)

Accordingly, the OCTA connectivity loss is defined as:


$$\begin{array}{l} L_{conn}^{\text{OCTA}}=1-\text{clDice}. \end{array}$$ (7)

This term encourages the predicted vessel probability map to preserve centerline continuity and suppress fragmentation of thin vessels. Thus, $L_{conn}$ in OCTA serves as a generic continuity prior, reducing vessel breaks caused by noise, low contrast, or projection artifacts.

For simplicity, the final connectivity term is denoted uniformly as $L_{conn}$, with its concrete form determined by the task.

#### 3.4.4 Training objective

Combining the above structural terms with the voxel-wise supervision term $L_{pixel}$, the total training objective is defined as:


$$\begin{array}{l} L_{total}=\alpha \cdot L_{pixel}+\lambda_{1}\cdot L_{exist}+\lambda_{2}\cdot L_{conn}, \end{array}$$ (8)

where $L_{pixel}$ denotes the voxel-wise segmentation loss, $L_{exist}$ denotes the existence-level structural supervision, and $L_{conn}$ denotes the connectivity-level structural supervision. The coefficients α, λ_1_, and λ_2_ are weighting factors used to balance voxel-level accuracy and structure-level regularization. This unified-yet-task-specific formulation enables GA-TAN to produce predictions that are not only voxel-wise accurate but also structurally coherent and anatomically plausible across both OCTA and MRA.

## 4 Experiments

### 4.1 Datasets

#### 4.1.1 OCTA dataset Retina3D

We evaluate 3D retinal vessel segmentation on two OCTA datasets acquired from different imaging platforms, referred to as Retina3D-Optovue and Retina3D-Zeiss. Retina3D-Zeiss consists of 34 volumetric OCTA scans (including both healthy subjects and diabetic retinopathy cases), while Retina3D-Optovue contains 25 healthy volumetric OCTA scans. All scans cover a 3 mm × 3 mm retinal region with volumetric depth information. For both datasets, 3D vessel annotations were provided by experienced ophthalmologists following a consistent annotation protocol and are used as ground truth for supervised training and evaluation. We evaluate Retina3D under a binary vessel-vs.-background segmentation protocol.

#### 4.1.2 MRA dataset TopCow

To evaluate the performance of the proposed model, we performed experiments on public dataset TopCow (7). The TopCow dataset includes 125 MRI images, each with dimensions of 185 × 508 × 585 and annotated with 13 arterial categories, which we treat as a multi-class semantic vessel segmentation task.

### 4.2 Implementation details

The GA-TAN was implemented in Python using the PyTorch library. The experiments were conducted on two separate NVIDIA GPUs (GeForce GTX 4090, 24 GB). We employed a 3D input with a patch size of 96 × 120 × 120. To ensure fairness, we evenly distribute the various topological variations in the dataset, allocating different types of topological variations into the training, validation, and test sets in a 6:2:2 ratio. We used the Adam optimizer with an initial learning rate of 0.01, a batch size of 2, and 1,000 training epochs. The weighted parameters in the final loss function were set as: λ_1_ = 0.7, λ_2_ = 4, α = 1.

### 4.3 Evaluation metrics

We evaluate GA-TAN on two settings with different label spaces: (i) binary retinal vessel segmentation on Retina3D (vessel vs. background), and (ii) segment-wise multi-class cerebral vessel segmentation on TopCow with 13 arterial categories. Accordingly, we adopt metrics tailored to each setting.

#### 4.3.1 Retina3D (OCTA, binary segmentation)

To comprehensively assess retinal vessel segmentation performance, we report four complementary metrics. (1) *Dice Similarity Coefficient (DSC)* is used as the primary overlap metric:


$$\begin{array}{l} \text{DSC}=\frac{2|P\cap G|}{\left|P|+|G\right|}, \end{array}$$ (9)

where P and G denote the predicted and ground-truth vessel sets, respectively. (2) *Precision (PRE)* is reported to measure the reliability of positive predictions, which is particularly relevant for thin vessels that are prone to false positives:


$$\begin{array}{l} \text{PRE}=\frac{\text{TP}}{\text{TP}+\text{FP}}. \end{array}$$ (10)

(3) *Centerline Dice (clDice)* is adopted to evaluate topological integrity by comparing the overlap of skeletonized centerlines, making it sensitive to connectivity preservation in fine capillary structures. (4) *Average Symmetric Surface Distance (ASD)* is used as a boundary-based metric to quantify the mean symmetric distance between the predicted and ground-truth vessel surfaces; lower ASD indicates better boundary accuracy.

#### 4.3.2 TopCow (MRA, 13-class segmentation)

For TopCow, we follow the official protocol and use the *macro-average Dice* over all *N* = 13 arterial categories. Let class *c* be treated as the positive class. The class-wise Dice is computed as:


$$\begin{array}{l} \text{DS}{\text{C}}_{c}=\frac{2\text{T}{\text{P}}_{c}}{2\text{T}{\text{P}}_{c}+\text{F}{\text{P}}_{c}+\text{F}{\text{N}}_{c}}, \end{array}$$ (11)

where TP_*c*_, FP_*c*_, and FN_*c*_ denote true positives, false positives, and false negatives for class *c*, respectively. The final score is obtained by macro-averaging across classes:


$$\begin{array}{l} \text{DS}{\text{C}}_{\text{mean}}=\frac{1}{N}\overset{N}{\underset{c=1}{\sum }}\text{DS}{\text{C}}_{c}. \end{array}$$ (12)

This metric equally weights all arterial categories and thus reflects overall segment-wise performance under the multi-class setting.

### 4.4 Comparison with state-of-the-art

In order to comprehensively evaluate the performance of the proposed network for blood vessel segmentation in eye OCTA modality and brain MRA modality, we conducted extensive experiments on the two datasets and also tested the performance of the world's mainstream segmentation networks on these two datasets.

The compared methods span three representative paradigms to ensure a comprehensive evaluation. For CNN-based approaches, we include UNet++ (34) and nnUNet (16), which are widely adopted baselines for volumetric medical image segmentation and excel at capturing local vascular features through hierarchical convolutions. For pure Transformer architectures, we select UNETR (17) and nnFormer (35), which leverage self-attention mechanisms to model long-range dependencies across the entire volume. For hybrid CNN–Transformer methods, we compare with Swin UNETR (18), Phtrans (36), Vsmtrans (37), and HmsUnet (38), which combine local feature extraction with global context modeling to balance detail preservation and holistic understanding. This selection covers the mainstream design spectrum and allows us to systematically analyze the strengths and limitations of each paradigm for vascular segmentation.

**Figure 4:**
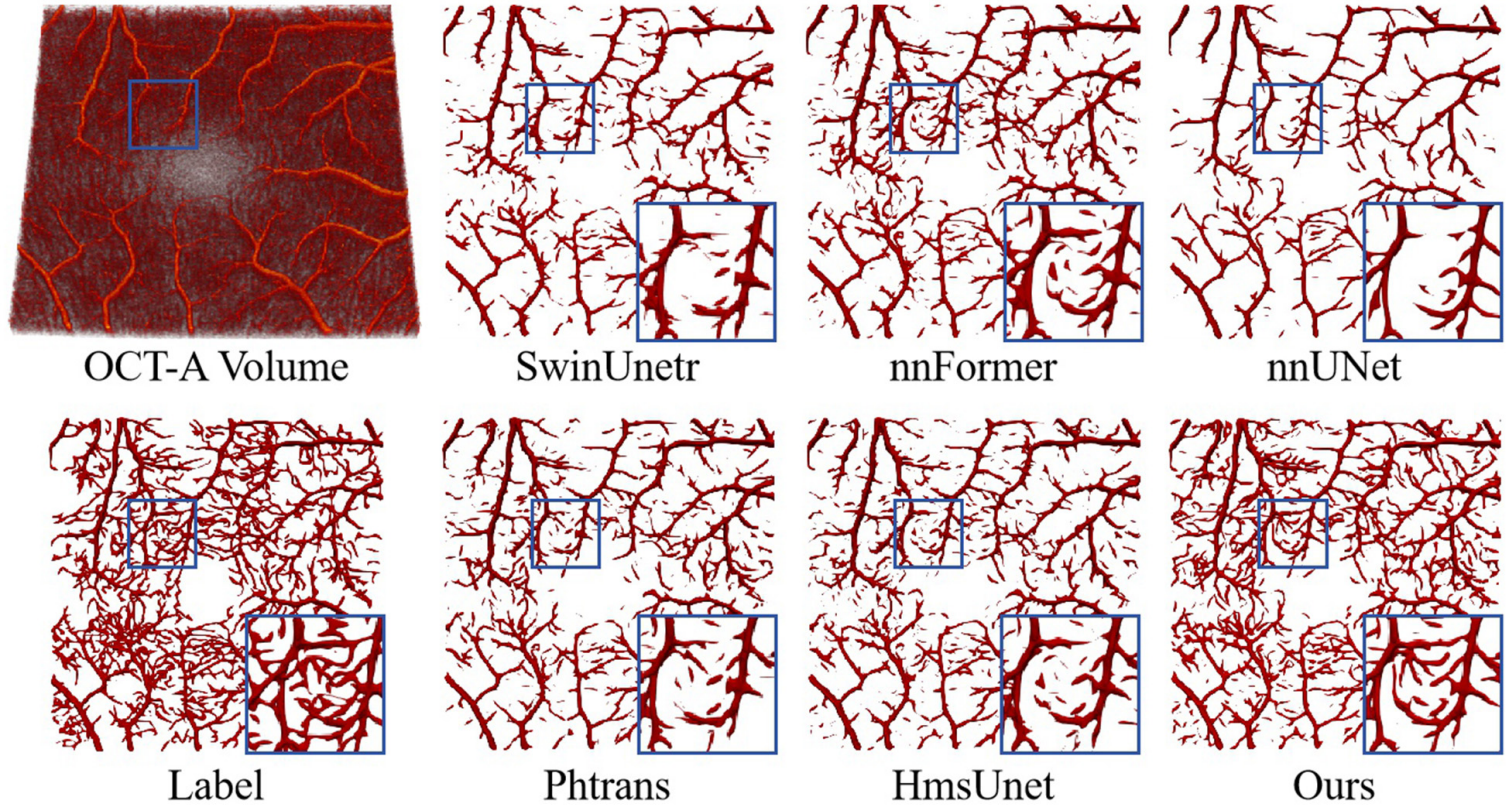
Direct comparison of the performance of different methods for vascular segmentation on the Retina3D dataset.

For the 3D retinal vessel segmentation task on the Retina3D dataset, results are presented in Table 1. Our method achieves the highest DSC of 0.684 and clDice of 0.667, surpassing the second-best methods nnFormer and nnUNet by 0.7 and 0.6 percentage points in DSC and clDice, respectively. The improvement in clDice is particularly noteworthy, as this metric directly reflects the preservation of vessel centerline connectivity, which is critical for accurate retinal vascular topology analysis. Compared with other hybrid methods such as HmsUnet (DSC 0.670, clDice 0.657) and Phtrans (DSC 0.670, clDice 0.657), our method consistently achieves higher scores, with improvements of 1.4 and 1.0 points in DSC and clDice, respectively. We note that ASD does not fully align with DSC/clDice in Retina3D. Since ASD measures boundary distance, some methods may obtain relatively low ASD by producing smoother or more conservative predictions on major vessels, even if they miss many thin capillaries. In contrast, DSC and especially clDice are more sensitive to the completeness and continuity of the retinal vascular network, which better reflects the practical goal of thin-vessel segmentation in this dataset. The qualitative comparisons in Figure 4 further support the quantitative results. Compared with other methods, the proposed GA-TAN produces more continuous and complete vascular structures on Retina3D, especially in thin vessels, branching regions, and locally complex areas. As highlighted in the zoomed-in regions, competing methods tend to exhibit vessel discontinuities, missing fine branches, or inaccurate structural recovery, whereas our method preserves vascular connectivity and topology more faithfully while reducing fragmentation. These results indicate that GA-TAN is more advantageous for 3D OCTA retinal vessel segmentation, yielding more accurate and anatomically coherent vessel delineation.

**Table 1:** Performance comparison of the proposed network and different mainstream segmentation networks on the Retina3D dataset.

Method	DSC↑	PRE↑	clDice↑	ASD↓
SwinUnetr (18)	0.620 ± 0.061	0.745 ± 0.126	0.602 ± 0.094	4.152 ± 1.297
Unetr (17)	0.449 ± 0.042	0.248 ± 0.037	0.401 ± 0.061	**2.978**±1.043
HmsUnet (38)	0.670 ± 0.034	0.682 ± 0.051	0.657 ± 0.064	4.213 ± 0.733
nnUNet (16)	0.578 ± 0.044	0.451 ± 0.071	0.661 ± 0.049	3.280 ± 1.016
nnFormer (35)	0.677 ± 0.042	0.754 ± 0.048	0.653 ± 0.059	3.090 ± 0.538
Vsmtrans (37)	0.653 ± 0.072	0.738 ± 0.062	0.657 ± 0.060	3.585 ± 1.001
Phtrans (36)	0.670 ± 0.034	0.682 ± 0.051	0.657 ± 0.064	4.352 ± 1.431
**Ours**	**0.684**±0.042	**0.749**±0.062	**0.667**±0.060	3.213 ± 0.538

Bold values indicate the best performance in each column.

To further assess robustness across acquisition sources, we additionally report source-wise results on Retina3D-Optovue and Retina3D-Zeiss in Table 2. On both subsets, the proposed method achieves the best DSC, PRE, and clDice among the compared methods, indicating that its advantage is consistent across different OCTA acquisition sources. Specifically, on Retina3D-Optovue, GA-TAN achieves a DSC/clDice of 0.701/0.684, outperforming nnFormer (0.693/0.668), and HmsUnet (0.684/0.671). On Retina3D-Zeiss, GA-TAN also remains superior, with a DSC/clDice of 0.672/0.654, compared with 0.665/0.642 for nnFormer and 0.660/0.647 for HmsUnet. Although the absolute performance on Zeiss is slightly lower than that on Optovue for all compared methods, the relative ranking remains stable, suggesting that the proposed framework preserves its effectiveness under scanner-dependent appearance variations. In addition, similar to the overall Retina3D results, ASD does not fully align with overlap and connectivity metrics, so the source-wise comparison mainly supports robustness from the perspectives of overlap accuracy and vascular continuity.

**Table 2:** Source-wise analysis on Retina3D.

Method	Retina3D-Optovue				Retina3D-Zeiss			
	DSC↑	PRE↑	clDice↑	ASD↓	DSC↑	PRE↑	clDice↑	ASD↓
nnFormer	0.693	0.768	0.668	**2.864**	0.665	0.744	0.642	**3.256**
HmsUnet	0.684	0.701	0.671	3.874	0.660	0.668	0.647	4.462
**Ours**	**0.701**	**0.764**	**0.684**	2.986	**0.672**	**0.738**	**0.654**	3.380

We report segmentation performance separately on Retina3D-Optovue and Retina3D-Zeiss to evaluate robustness across OCTA acquisition sources. Bold values indicate the best performance in each column.

**Figure 5:**
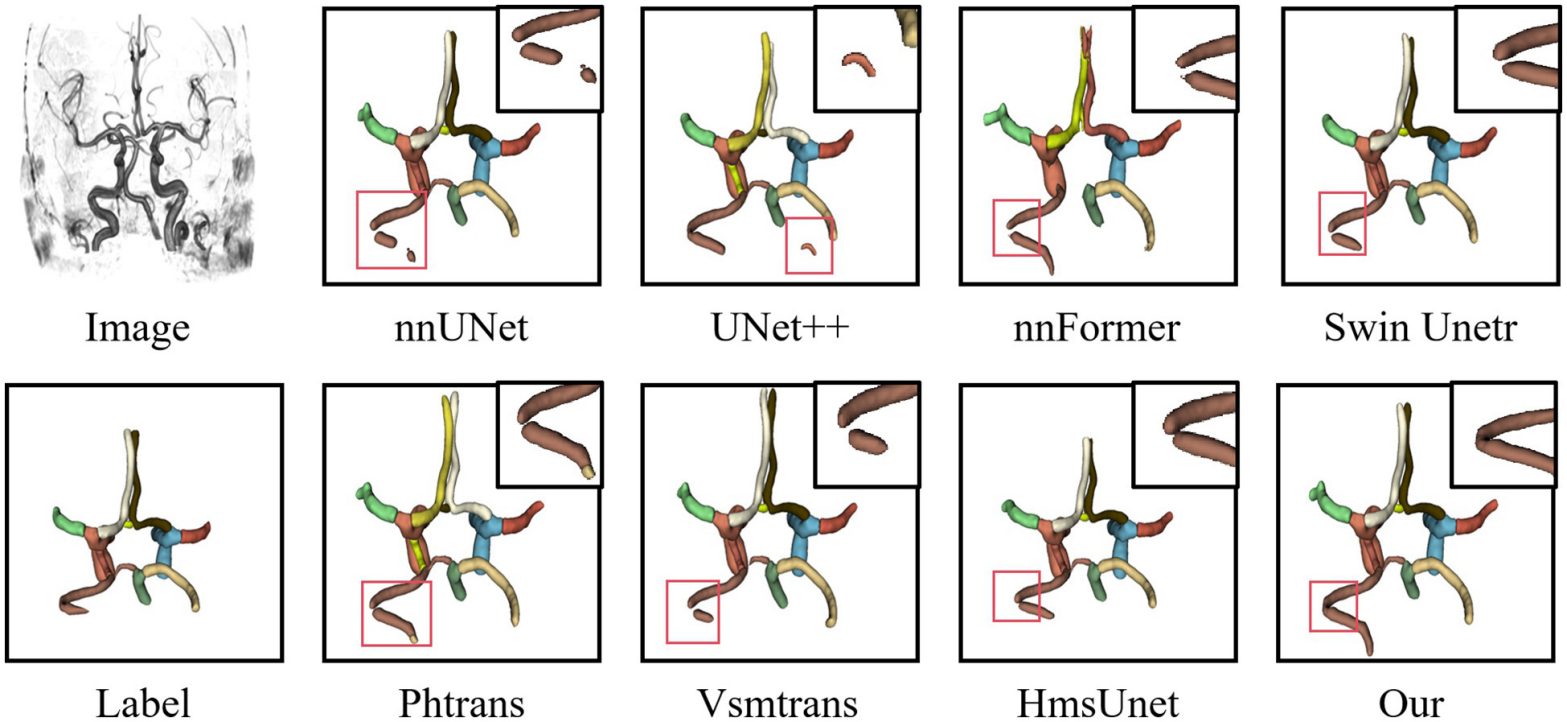
Direct comparison of the performance of different methods for vascular segmentation on the TopCow dataset. The red box represents the prediction error.

For multi-class CoW vessel segmentation on the TopCow dataset, quantitative results are reported in Table 3. Our method achieves a mean Dice score of 81.1%, outperforming the best competing method HmsUnet by 1.0 percentage point. Compared with CNN-based methods, our approach surpasses nnUNet and UNet++ by 1.1 and 2.7 points, respectively, indicating that purely convolutional architectures are limited in modeling long-range topological relationships between distant vessel segments. Among pure Transformer methods, nnFormer achieves only 61.8% due to insufficient sensitivity to fine vascular details, while UNETR obtains 58.5%, suggesting that global self-attention alone cannot adequately capture the local geometric characteristics of thin vessels. Our method also outperforms hybrid approaches including SwinUnetr (79.5%), Vsmtrans (78.5%), and Phtrans (74.6%) by 1.6, 2.6, and 6.5 points, respectively. Notably, our method achieves the best performance on 6 out of 13 vessel categories, including PCA-L (0.847), MCA-L (0.805), ICA-R (0.872), Pcomm-R (0.806), ACA-L (0.782), and ACA-R (0.815), demonstrating its superior ability to handle both large arterial trunks and thin communicating arteries. Qualitative results in Figure 5 further confirm that our method produces more anatomically consistent segment-wise multi-class segmentation with fewer topological errors. These improvements can be attributed to the multi-scale graph aggregation module, which effectively mitigates the class imbalance between large and small vessel segments, and the topology-aware loss, which explicitly supervises vessel connectivity. These results demonstrate that the proposed GA-TAN, through its variable window mechanism for multi-scale context modeling, efficient multi-scale attention for enhanced spatial–channel interaction, and graph aggregation for cross-level feature fusion, effectively captures both fine capillary details and global vascular topology, enabling more precise vessel segmentation across different imaging modalities.

**Table 3:** Performance comparison on TopCow for segment-wise multi-class CoW vessel segmentation.

Methods	BA	PCA		MCA		ICA		Pcomm		Acomm	ACA		3rd-a2	mean
		L	R	L	R	L	R	L	R		L	R	
UNet++ (34)	0.853	0.853	**0.850**	0.776	0.818	0.826	0.868	**0.878**	0.469	0.547	0.768	0.800	0.880	0.784
nnUNet (16)	0.872	0.763	0.783	0.791	**0.830**	0.786	0.864	0.843	0.785	**0.666**	0.763	0.780	0.880	0.800
nnFormer (35)	0.809	0.658	0.658	0.555	0.442	0.671	0.694	0.640	0.440	0.320	0.656	0.637	0.880	0.618
SwinUnetr (18)	0.864	0.780	0.803	0.764	0.821	0.797	0.853	0.771	0.782	0.610	0.765	0.786	**0.934**	0.795
Unetr (17)	0.829	0.715	0.657	0.701	0.659	0.757	0.705	0.403	0.342	0.433	0.659	0.588	0.151	0.585
Phtrans (36)	**0.891**	0.829	0.795	0.792	0.824	0.823	0.871	0.564	0.645	0.566	0.781	0.792	0.880	0.746
Vsmtrans (37)	0.866	0.778	0.694	0.773	0.801	**0.850**	0.861	0.767	0.711	0.602	0.767	0.799	0.880	0.785
HmsUnet (38)	0.862	0.826	0.795	0.732	0.812	0.810	0.864	0.830	0.827	0.636	0.763	0.777	0.880	0.801
Proposed	0.879	**0.847**	0.835	**0.805**	0.805	0.824	**0.872**	0.775	**0.806**	0.618	**0.782**	**0.815**	0.875	**0.814**

We report Dice for each arterial category and the mean Dice across 13 categories, following the TopCow evaluation protocol. The “L” means Left and the “R” means Right. Bold values indicate the best performance in each column.

### 4.5 Ablation study

Tables 4, 5 present the ablation results on the Retina3D and TopCow datasets, respectively, to evaluate the contribution of each proposed component. The baseline (BB) refers to the hybrid CNN–Transformer backbone with the variable window structure.

**Table 4:** Ablation results for 3D retinal vessel segmentation on the Retina3D dataset.

Method	DSC↑	PRE↑	clDice↑	ASD↓
BB	0.568 ± 0.531	0.612 ± 0.392	0.539 ± 0.588	**3.021**±1.592
BB + EMA	0.601 ± 0.295	0.642 ± 0.502	0.593 ± 0.402	3.423 ± 1.235
BB + MSGA	0.621 ± 0.357	0.659 ± 0.392	0.630 ± 0.349	3.593 ± 0.932
BB + ALL	0.653 ± 0.213	0.710 ± 0.091	0.632 ± 0.121	3.847 ± 1.032
BB + ALL + Topo	**0.684**±0.042	**0.749**±0.062	**0.667**±0.060	3.213 ± 0.538

Bold values indicate the best performance in each column.

**Table 5:** Ablation results for 3D brain vessel segmentation on the TopCow dataset.

Methods	BA	PCA		MCA		ICA		Pcomm		Acomm	ACA		3rd-a2	mean
		L	R	L	R	L	R	L	R		L	R	
BB	0.833	0.764	0.761	0.764	0.758	0.860	0.857	0.772	0.738	0.645	0.768	0.774	0.880	0.783
BB + EMA	**0.897**	0.842	0.816	0.787	0.778	**0.864**	0.869	0.682	0.658	0.607	**0.802**	0.787	0.880	0.790
BB + MSGA	0.872	0.824	0.798	**0.813**	0.807	0.851	0.867	0.813	**0.832**	0.510	0.789	0.778	0.880	0.803
BB + ALL	0.879	**0.847**	**0.835**	0.805	0.805	0.824	**0.872**	0.775	0.806	0.618	0.782	**0.815**	0.875	0.811
BB + ALL + Topo	0.880	0.821	0.795	0.803	**0.854**	0.803	0.853	**0.835**	0.793	**0.683**	0.781	0.795	**0.912**	**0.814**

“BB” denotes the backbone, and “Topo” denotes the topology-aware objective. Bold values indicate the best performance in each column.

#### 4.5.1 Effect of the EMA module

On the Retina3D dataset, incorporating the EMA module (BB + EMA) improves DSC from 0.568 to 0.601 (+3.3 points) and clDice from 0.539 to 0.593 (+5.4 points). On the TopCow dataset, the EMA module raises the mean Dice from 78.3% to 79.0% (+0.7 points), with particularly notable improvements on BA (+6.4 points, from 0.833 to 0.897) and ACA-L (+3.4 points, from 0.768 to 0.802). As described in Section 3, the EMA module establishes joint spatial–channel attention through coordinate attention along three orthogonal axes, enabling the capture of long-range cross-channel dependencies and 3D global contextual features. This mechanism is especially beneficial for enhancing the spatial representation of vessels with complex 3D orientations, such as the basilar artery and anterior cerebral arteries in MRA, and fine capillary structures in OCTA.

#### 4.5.2 Effect of the MSGA module

The MSGA module yields a more substantial improvement. On the Retina3D dataset, BB + MSGA achieves a DSC of 0.621 (+5.3 points over BB) and clDice of 0.630 (+9.1 points), indicating that graph-based multi-level feature fusion significantly enhances vessel centerline connectivity. On the TopCow dataset, the MSGA module improves the mean Dice to 80.3% (+2.0 points), with the most pronounced gains on Pcomm-R (+9.4 points, from 0.738 to 0.832) and MCA-L (+4.9 points, from 0.764 to 0.813). These thin communicating and middle cerebral arteries are precisely the vessel categories that suffer most from scale imbalance. Through its hierarchical graph convolution and cross-layer feature concatenation embedded in skip connections, the MSGA module effectively promotes multi-scale information integration and mitigates the class imbalance between large arterial trunks and small vessel segments.

#### 4.5.3 Effect of combined modules

When both EMA and MSGA are integrated (BB + ALL), the performance further improves. On the Retina3D dataset, DSC reaches 0.653 (+8.5 points over BB) and PRE reaches 0.710 (+9.8 points). On the TopCow dataset, the mean Dice increases to 81.1% (+2.8 points over BB), achieving the best scores on PCA-L (0.847), PCA-R (0.835), ICA-R (0.872), and ACA-R (0.815). The complementary nature of the two modules is evident: EMA strengthens spatial–channel feature representation while MSGA enhances cross-scale graph-based feature fusion, and their combination achieves synergistic improvements that exceed the sum of individual contributions.

#### 4.5.4 Effect of the topology loss

Finally, adding the topology-aware objective (BB + ALL + Topo) achieves the best overall performance on both datasets. On the Retina3D dataset, DSC further improves to 0.684 (+3.1 points over BB + ALL) and clDice to 0.667 (+3.5 points), with a notably reduced standard deviation (from ±0.213 to ±0.042 in DSC), indicating more stable and consistent segmentation. On the TopCow dataset, the mean Dice reaches 81.4%, with significant improvements on topologically challenging categories: Pcomm-L increases from 0.775 to 0.835 (+6.0 points), Acomm from 0.618 to 0.683 (+6.5 points), and 3rd-a2 from 0.875 to 0.912 (+3.7 points). These categories correspond to communicating arteries and variant segments that are most susceptible to topological disruptions. By explicitly supervising vessel existence and connectivity, the topology-aware objective effectively reduces segmentation errors arising from anatomical variants in MRA and noise-induced breaks in OCTA, thereby enhancing the topological correctness of the predicted vascular structures.

## 5 Conclusion and future work

In this work, we have presented GA-TAN, a unified graph aggregation and topology-aware framework for 3D vascular binary retinal and multi-class cerebral vessel segmentation across OCTA and MRA. Motivated by the shared challenges in ocular and cerebral vascular imaging—including extreme multi-scale morphology and topology disruptions induced by imaging artifacts or anatomical variants—GA-TAN has integrated three complementary designs: a variable-window hybrid backbone for adaptive multi-scale context modeling, an efficient multi-scale attention module to strengthen volumetric spatial–channel interactions, and a multi-scale graph aggregation module for global topological reasoning and cross-level feature fusion. In addition, we have introduced a topology-aware training objective that explicitly supervises vessel existence and connectivity, encouraging anatomically plausible and topologically consistent predictions. Extensive experiments on Retina3D and TopCow have demonstrated that GA-TAN has consistently outperformed representative CNN-based, Transformer-based, and hybrid baselines, especially in preserving vascular continuity and improving robustness on thin/short vessel segments.

Future work will proceed in several directions. First, we will evaluate the generalization capability of GA-TAN under broader real-world settings, including multi-center data, different scanners, and diverse acquisition protocols, where domain shift is often substantial for both OCTA and MRA. Second, to alleviate the reliance on dense 3D annotations, we plan to explore semi-supervised and weakly-supervised learning strategies, such as consistency regularization, pseudo-labeling, and topology-guided self-training, to exploit large-scale unlabeled volumes more effectively. Third, we will further refine the topology modeling by incorporating more explicit anatomical priors (e.g., modality-specific adjacency constraints and variant-aware rules) and investigating more principled topological objectives that balance connectivity enforcement with tolerance to true anatomical absence. Finally, we aim to extend the framework toward clinically actionable analysis by coupling segmentation with downstream vascular biomarker extraction and cross-modality association studies, enabling more reliable quantitative assessment of eye–brain vascular phenotypes.

## Data Availability

The original contributions presented in the study are included in the article/supplementary material, further inquiries can be directed to the corresponding authors.
